# Identification of the gene signatures related to NK/T cell communication to evaluate the tumor microenvironment and prognostic outcomes of patients with prostate adenocarcinoma

**DOI:** 10.3389/fimmu.2025.1564784

**Published:** 2025-04-16

**Authors:** Kun Zhang, Huyang Xie, Fan Zhao, Yeqing Huang

**Affiliations:** Department of Urology, Affiliated Hospital of Nantong University, Nantong, China

**Keywords:** NK/T cell communication, tumor microenvironment, prostate cancer, immune escape, machine learning

## Abstract

**Background:**

Prostate adenocarcinoma (PRAD) is a leading cause of male mortality, with NK/T cell communication being key areas of the research.

**Methods:**

The Seurat package was utilized to normalize and reduce the dimensionality of the single-cell data, and CellMarker 2.0 was employed for cell annotation. CellChat was utilized to construct the ligand-receptor interaction network of cell subsets. Differentially expressed genes (DEGs) were filtered by the limma package. Univariate Cox and the LASSO regression in the glmnet package were used to obtain biomarkers and develop a risk model. The survminer package was used to calculate the optimal threshold for dividing patients into high-risk and low-risk groups, and then Kaplan-Meier (KM) survival analysis was performed. Single-sample GSEA (ssGSEA), TIMER, and ESTIMATE packages were employed for immune infiltration analysis. Pathway analysis was conducted for the low- and high-risk groups using GSEA. Immunotherapy responses were evaluated by adopting TIDE method. Additional cellular validation (quantitative real-time PCR, CCK-8, Transwell, and scratch assay) was implemented to confirm the effects of feature genes on PRAD.

**Results:**

Compared with the benign group, NK/T cells were the cell type with the greatest changes in the tumor group, and their communication intensity was relatively high among all cell types. A RiskScore model was constructed as follows: 0.579*FOXS1 + 0.345*GPC6 + 0.385*ISYNA1 + 0.418*ITGAX + 0.792*MGAT4B + 0.368*PRR7 + 0.458*REXO2 . Analysis of the differences between the two risk groups showed that the level of immune infiltration was higher in the high-risk group, and it was significantly enriched in immune-correlated pathways, while the low-risk group was mainly enriched in metabolism-related pathways. TIDE analysis indicated that the high-risk group had higher immune escape potential. The cellular validation assays have revealed the higher expression of seven biomarkers in PRAD groups. Further, *ISYNA1* knockdown inhibited the proliferation, migration, and invasion ability of PRAD cells.

**Conclusion:**

The current research reveals key communication genes in PRAD, offering new possibilities for the exploration of new therapeutic targets.

## 1 Introduction

Globally, prostate cancer (PCa) is a common cause of death among men. Statistics show that in 2022, there were 1,466,680 new PCa cases globally, accounting for 7.3% of new cancers and ranking fourth (1). More than 95% of PCa cases are adenocarcinomas, with the majority originating from acini and a minority originating from ducts (2). Its incidence is closely related to location and age (3, 4). In the early stage, prostate adenocarcinoma (PRAD) has relatively mild symptoms, with the most common being dysuria and increased frequency of urination (5). In the advanced stage, PRAD may present with urinary retention and back pain (5). Currently, the main treatment method for PCa is androgen deprivation therapy, but it is difficult to completely cure metastatic castration-resistant prostate cancer (mCRPC) caused by cancer metastasis (6). The research on immunotherapy for PRAD mainly focuses on Sipuleucel-T and monoclonal antibodies (7), but experiments have found that their efficacy in PRAD patients is not yet significant (8, 9). Given the long natural course of PRAD and the relatively high mortality rate of moderate or high-risk local, locally advanced or metastatic cancers (10), it is necessary to establish a prognostic model related to PRAD, which helps in the stratified treatment of patients and the selection of medical regimens.

The single-cell RNA sequencing (scRNA-seq) technique has offered unprecedented opportunities for studying intercellular interactions within the tumor microenvironment (TME) in recent years (11), particularly in the research of immune cells such as T cells and natural killer (NK) cells. NK/T cells play pivotal roles in tumor immune evasion and immune surveillance (12). Studies have found that combinations of NK-cell-based immunotherapy and T-cell-based immunotherapy may be beneficial for tumor immunotherapy (13). Nguyen et al. suggest that further detection of tumour-infiltrating lymphocytes in prostate cancer may help to gain insight into the regulatory mechanisms of T cells in the TME (14). In addition, NK/T cells interact with tumor cells and other immune cells through cellular communication (15). Currently, the systematic inference of ligand-receptor-mediated intercellular communication has become a research hotspot, which is closely linked to tumorigenesis (16, 17). Understanding these intercellular communication mechanisms can uncover new immune escape pathways and provide novel strategies for immunotherapy in PRAD.

In this study, by analyzing single-cell data from PRAD, we identified the cell communication receptor-ligand genes between NK/T cells and other cell types, and constructed their interaction networks. Through bioinformatics methods, we screened for differential genes related to the communication receptor-ligands between NK/T cells and other cells. This study developed a risk model with these key genes and conducted prognostic analysis. Then, we performed *in vitro* experimental verification on the obtained biomarkers and evaluated the immune infiltration levels and immune therapy responses of different risk groups. These findings not only provide potential biomarkers for early detection and prognostic assessment of PRAD but also open up new possibilities for future stratified and personalized treatment developments in PRAD.

## 2 Material and methods

### 2.1 Collection and processing of data

The gene expression data and related clinical data of PRAD patients were obtained from the The Cancer Genome Atlas (TCGA, https://portal.gdc.cancer.gov) database (18). The FPKM values of TCGA-PRAD RNA sequencing (RNA-Seq) data was transformed into TPM and then log2-converted. After retaining the samples with complete progression-free interval (PFI), 495 PRAD samples and 52 adjacent normal control samples were recruited. The expression data and clinical data (MSKCC, Cancer Cell 2010) of PRAD were extracted from the cBioPortal website (https://www.cbioportal.org/). After screening, a sum of 131 tumor samples were included. From Gene Expression Omnibus (GEO, https://www.ncbi.nlm.nih.gov/geo/), we sourced four benign and four PRAD single-cell samples in GSE193337. The library construction platform was 10x Genomics, and the sequencing platform was Illumina HiSeq 4000.

### 2.2 ScRNA-seq analysis

The Seurat object was created by using the CreateSeuratObject function in the Seurat package (19). Cells with the number of retained genes ranging from 200 to 8000 and the quantity of mitochondrial genes less than 20% were reserved. Next, the NormalizeData function (19) was used for normalization. After the principal component analysis (PCA) dimensionality reduction, batch effect was eliminated by the harmony package (20). Then, the RunUMAP function (19) was utilized for dimensionality reduction by uniform manifold approximation and projection (UMAP). Finally, cells were clustered by the FindNeighbors and FindClusters functions (19) with the parameters of dims = 1:30 and resolution = 0.1. According to the marker genes provided by the CellMarker2.0 database, the cell types were annotated (21).

### 2.3 Cell communication

CellChat (22) was used to develop the ligand-receptor interaction network of cell subpopulations. The netVisual_bubble and netVisual_circle packages (22) were utilized to display the bubble plots of receptors and ligands between NK/T cells and other cells and the number of communications between them.

### 2.4 Screening of differently expressed genes differentially expressed genes (DEGs)

Differential analysis between the tumor and control sample groups in the TCGA cohort was performed in the limma package (23). The screening criteria were that |log 2fold change (FC)| > log2(1.5) and *p* < 0.05, thus obtaining the DEGs. Then, the intersection was taken between the mRNAs related to the receptor and ligand genes of the communication between NK/T cells and other cells (cor > 0.5, *p* < 0.01) and the TCGA DEGs to obtain the mRNAs related to the communication of NK/T cells.

### 2.5 Development of the risk model and validation

The TCGA-PRAD samples were assigned randomly at the ratio of 5:5 into training set and test set. The survival package (24) was utilized to conduct univariate Cox regression analysis to determine the prognostic relevance of the intersection genes and *p* < 0.05 was selected for filtering. Subsequently, the glmnet package (25) was employed to carry out LASSO COX regression analysis and stepwise regression to further narrow down the gene scope. Through multivariate analysis, the key genes and their corresponding coefficients were calculated, and the RiskScore for patients was calculated based on the formula:


RiskScore = Σβi × Expi

where βi refers to the regression coefficient of each key gene, and Expi represents the expression of the corresponding gene. Then, the survminer package (26) was employed to calculate the optimal threshold to classify the patients into low- and high-risk groups. KM survival analysis was performed, and receiver operating characteristic (ROC) curve model was constructed to predict the prognostic performance of the TCGA training set and test set. The area under the curve (AUC) value is a robust metric for assessing the classification accuracy of the RiskScore model. By comparing the AUC values to the assessment criteria, we can determine the effectiveness of the model in distinguishing between high- and low-risk patients. To better validate whether the model was robust, the same method was employed to the MSKCC dataset for verification.

### 2.6 Analysis of the tumor immune microenvironment (TIME)

To analyze the association between RiskScore and the TIME, the infiltration status of immune cells was calculated using different methods. The ssGSEA function of GSVA (27, 28) was utilized to compute the scores of 28 types of tumor infiltrating immune cells (TIICs) (1). The TIMER online tool (http://cistrome.org/TIMER) was utilized to calculate six immune scores, and the ESTIMATE package (29) was used to score the immune cells.

### 2.7 Pathway enrichment analysis

The gene set enrichment analysis (GSEA) (30) was employed for pathway analysis to explore the pathways of diverse biological processes in the two risk groups. The candidate gene sets from the Kyoto Encyclopedia of Genes and Genomes (KEGG) database were used to conduct GSEA (31).

### 2.8 Evaluation of immunotherapy

The TIDE method was adopted to evaluate immunotherapy response. The standardized transcriptome data were uploaded into the TIDE website (http://tide.dfci.harvard.edu/) to calculate the TIDE score, with a higher TIDE prediction score indicating greater immune escape possibility and lower immunotherapy efficacy.

### 2.9 Cell culture

In this study, the cell lines, namely human normal prostate epithelial cells PNT1A (cat.no RE59812) and human PCa cells DU145 (cat.no YS101C), were sourced from Shanghai Yaji Biotechnology Co., Ltd (Shanghai, China) (http://www.yajimall.com/). All cells were cultured in Roswell Park Memorial Institute (RPMI) 1640 (11875093, Gibco, Grand Island, NY, USA) with the supplementation of 10% fetal bovine serum (FBS) (S9020, Solarbio Lifesciences, Beijing, China). All cells were incubated in the incubator at 37°C with 5% CO_2_. The cells underwent short tandem repeat (STR) identification, and the results of mycoplasma detection for these cells were found to be negative.

### 2.10 Quantitative real-time PCR

Following the instructions, total RNA was isolated from PNT1A and DU145 cells utilizing the TriZol total RNA extraction kit (15596026CN, Invitrogen, Carlsbad, CA, USA). Subsequently, the concentration of the isolated RNA was measured. Then, complementary DNA was synthesized by reverse transcription with a relevant assay kit (D7178S, Beyotime, Shanghai, China). After that, SYBR Green qPCR Mix (D7260, Beyotime, Shanghai, China) was used for the PCR assay according to the protocols. The conditions for qPCR included an initial step at 94°C for 30 seconds, succeeded by 40 cycles consisting of 5 seconds at 94°C and 30 seconds at 60°C. Finally, the relative level was calculated by the 2^-ΔΔCT^ method (32), with GAPDH as a reference gene. Based on National Center for Biotechnology Information (NCBI) sequences, the qRT-PCR primers used in this study were designed using Primer Premier 6 software. The primer sequences were presented in **Supplementary Table 1**.

### 2.11 Cell transfection

For the liposome transfection, the small interfering RNA against *ISYNA1* (si-*ISYNA1*) and the negative control small interfering RNA (si-NC) were all purchased from Merck KGaA (Shanghai, China) and transfected into DU145 cells utilizing lipofectamine 2000 transfection reagent (11668027, Invitrogen, Carlsbad, CA, USA) as per the manuals. The sequences applied for the transfection were 5’-GCACCCATCATGCTGGACCTA-3’(si-*ISYNA1*#1) and 5’-CAGAAGAATGGTACAAATCCAAG-3’(si-*ISYNA1*#2).

### 2.12 Cell viability

DU145 cells in the logarithmic growth phase were plated into a 96-well dish at a density of 1 × 10^4^ cells per well and incubated at 37°C with 5% CO_2_ for durations of 0, 24, 48, or 72 hours. Following this, 10 μL of CCK-8 reagent was added to each well, and the samples were incubated at 37°C for 2 hours. For the construction of the CCK-8 curve, absorbance measurements were taken at 450 nm, which served as the y-axis, while time was plotted on the x-axis.

### 2.13 Cell invasion assay

For the cell invasion assay, 1 × 10^5^ DU145 cells were suspended in 200 μL of serum-free medium and cultured in the upper chamber of the Transwell (3422, Corning, Inc., Corning, NY, USA) coated with Matrigel (C0372, Beyotime, China), while 700 μL of medium containing 10% bovine serum were supplemented to the lower Transwell chamber. After incubation, the chamber was taken out and gently rinsed with phosphate buffered saline (PBS) buffer (P1010, Solarbio Lifesciences, Beijing, China) to remove the non-invasive cells. Subsequently, 4% paraformaldehyde (P1110, Solarbio Lifesciences, Beijing, China) was employed for fixing the cells, which were dyed by 0.1% crystal violet (G1063, Solarbio Lifesciences, Beijing, China) at room temperature for 20 minutes. The invasive cells were observed with an optical microscope (DP27, Olympus, Tokyo, Japan) in three randomly selected fields of view (33). Finally, to ensure the accuracy and reproducibility of cell counting, we used ImageJ software for automated cell counting.

### 2.14 Cell migration assay

The DU145 cells (5 × 10^5^ cells/well), after being transfected, were grown in a 6-well plate containing media devoid of serum. Upon reaching full confluence, an artificial wound was introduced into the monolayer using a 200-μL sterile pipette tip. Following a 48-hour incubation period, the cells were photographed under an inverted optical microscope (DP27, Olympus, Japan). Subsequently, the percentage of wound closure (%) was calculated to reflect the migration ability of the PRAD cells, ensuring uniqueness in reporting (34).

### 2.15 Statistical analysis

All the statistical data were analyzed in R language (version 3.6.0). Experimental data were analyzed using GraphPad Prism 8.0 software. The Wilcoxon rank-sum test was used to compare the differences between two-group continuous variables, the Spearman method was employed to calculate the correlations, the log-rank test was utilized to compare the survival differences among patients in each grouping. Unpaired t-test and one-way analysis of variance were applied for the comparison on the experimental data, and *p* < 0.05 signified a statistical significance.

## 3 Results

### 3.1 Single-cell atlas of PRAD

**Figure 1:**
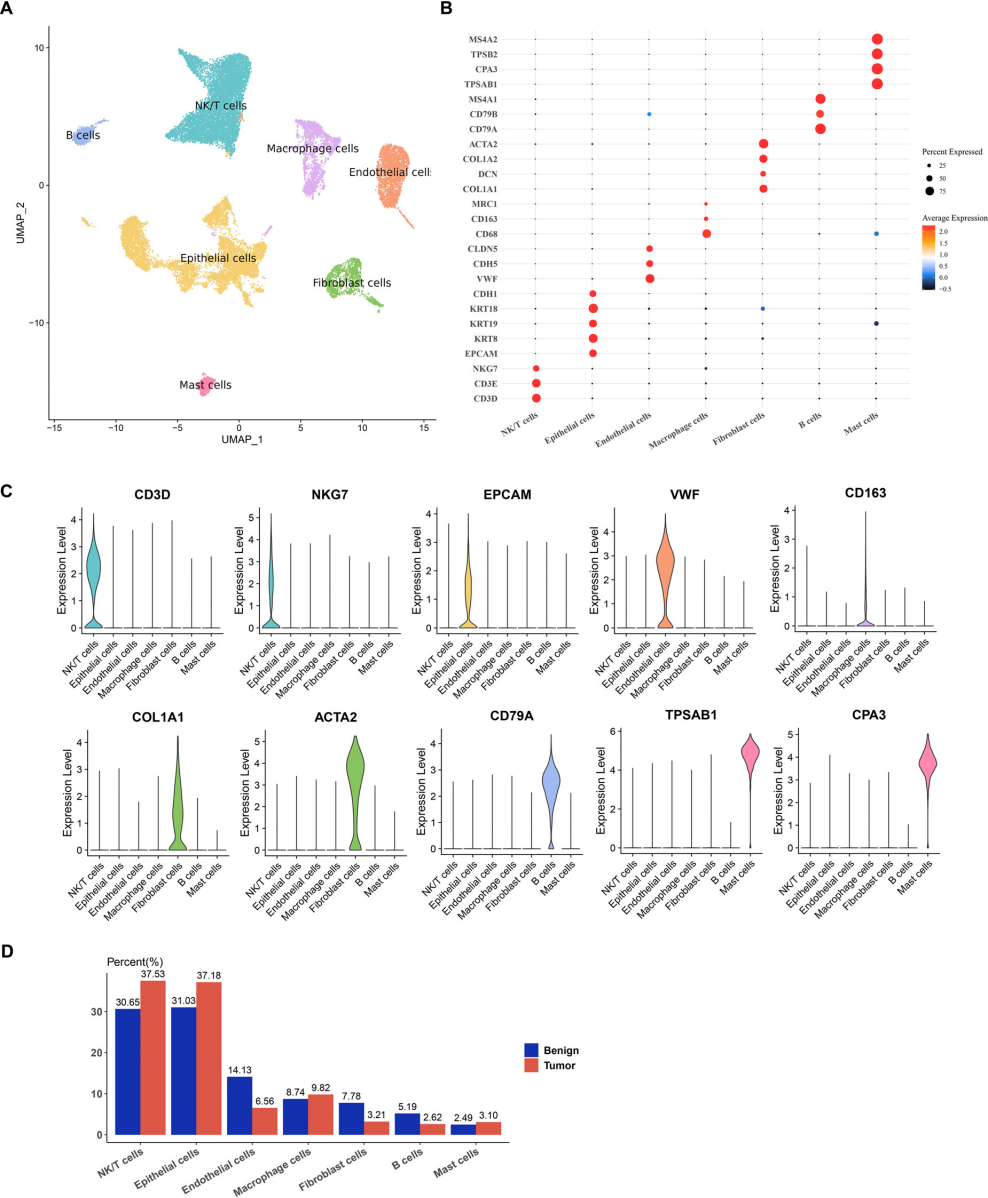
Single-cell atlas of PRAD. **(A)** UMAP dimensionality reduction plot for single-cell clustering and annotation of benign and tumor samples from PRAD. **(B)** Bubble plot for annotating the expression of marker genes in subpopulations. **(C)** Violin plot illustrating the expression of marker genes. **(D)** Proportion of each cell subpopulation within each sample between the two groups.

Single-cell clustering and annotation analysis were conducted on samples from benign and tumor tissues of PRAD, identifying a total of seven cell subpopulations: NK/T cells, Macrophage cells, Epithelial cells, Fibroblast cells, Mast cells, Endothelial cells, and B cells (**Figure 1A**). **Figures 1B, C** depict the marker genes for each of these cell subpopulations. Among them, high expression of *CD3D* and *NKG7* was observed in NK/T cells; *EPCAM* in epithelial cells; *VWF* in endothelial cells; *CD163* in macrophages; *COL1A1* and *ACTA2* in fibroblasts; *CD79A* in B cells; and *TPSAB1* and *CPA3* genes in mast cells. Analysis of the distribution of various cell types between the benign and tumor groups revealed that, compared to the benign group, NK/T cells exhibited the highest proportional increase in the tumor group (**Figure 1D**).

### 3.2 Signal communication among various cell types

**Figure 2:**
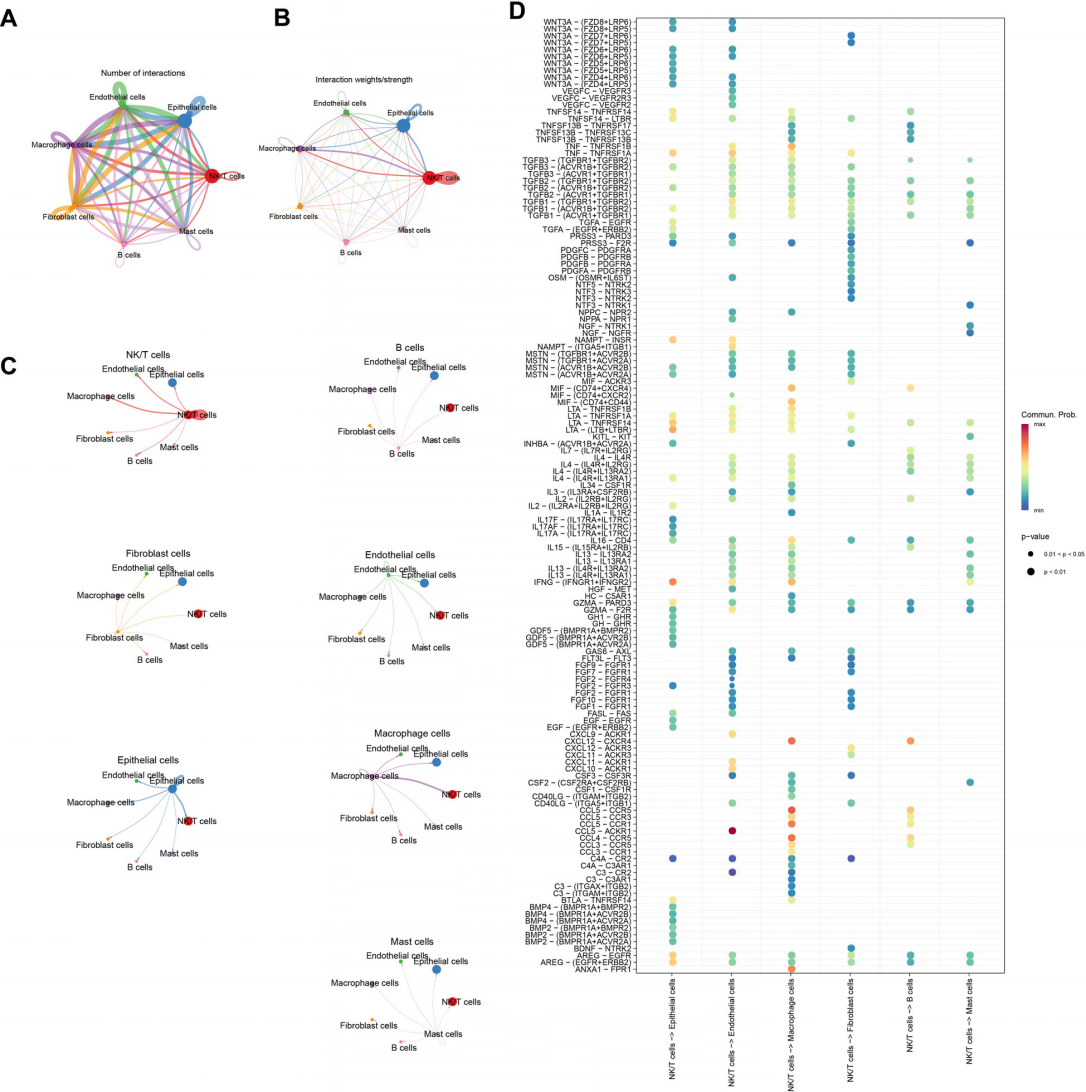
Analysis of signaling communication between NK/T Cells and other cell types. **(A)** Number of communications between cells. **(B, C)** Intensity of communications between cells. **(D)** Ligand-receptor mediated cellular communications between NK/T cells and other cell types.

Analysis of the number of communications among various cell types revealed that B cells had the least number of communications among all cell types (**Figure 2A**). Examination of the intensity of communications among cells showed that NK/T cells exhibited high communication intensity, indicating their active role in the TME (**Figure 2B**). To visually present this point more clearly, the communication intensity of each cell type was further detailed, and the results demonstrated that the communication intensity between NK/T cells and Macrophage cells, Epithelial cells, as well as Endothelial cells was particularly prominent (**Figure 2C**). These findings showed that NK/T cells played a pivotal role in immune regulation within the TME and have close interactions with other key cell types. **Figure 2D** further illustrates the receptors and ligands involved in cellular communications.

### 3.3 Identification of DEGs related to communication receptors and ligands

**Figure 3:**
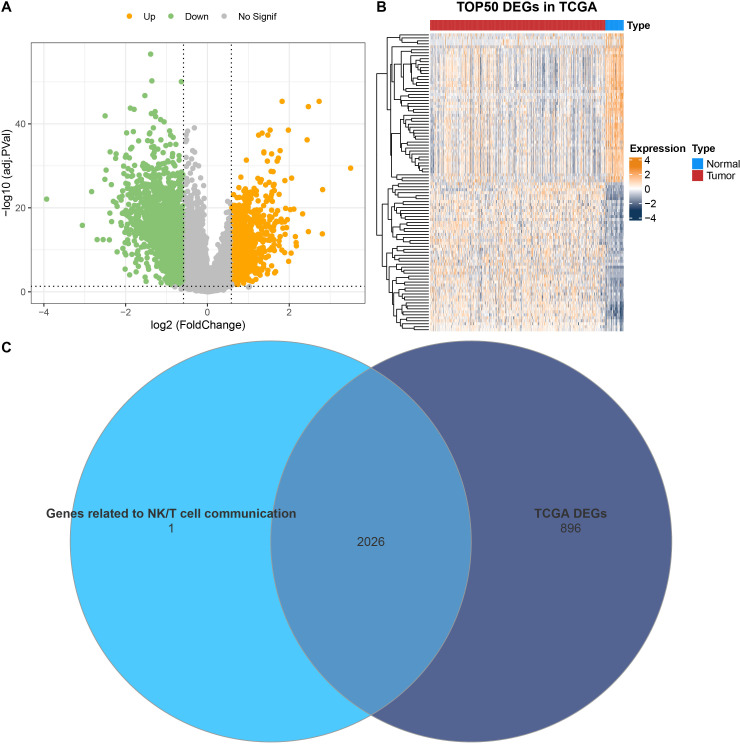
Identification of DEGs. **(A)** Volcano plot on the distribution of DEGs between PRAD and control samples in TCGA. **(B)** Heatmap illustrating the expression of the top 50 DEGs in TCGA. **(C)** Venn diagram showing the intersection between mRNAs related to communication receptors and ligands between NK/T cells and other cells, as well as the DEGs in TCGA.

Differential analysis was conducted between tumor and control sample groups in the TCGA cohort, resulting in 1951 downregulated genes and 971 upregulated genes (**Figures 3A, B**). Next, these DEGs were intersected with mRNAs linked to communication receptors and ligands between NK/T cells and other cells (correlation coefficient > 0.5, *p* < 0.01). This intersection identified a total of 2026 overlapping genes that are associated with communication between NK/T cells (**Figure 3C**).

### 3.4 Model construction and validation in PRAD

**Figure 4:**
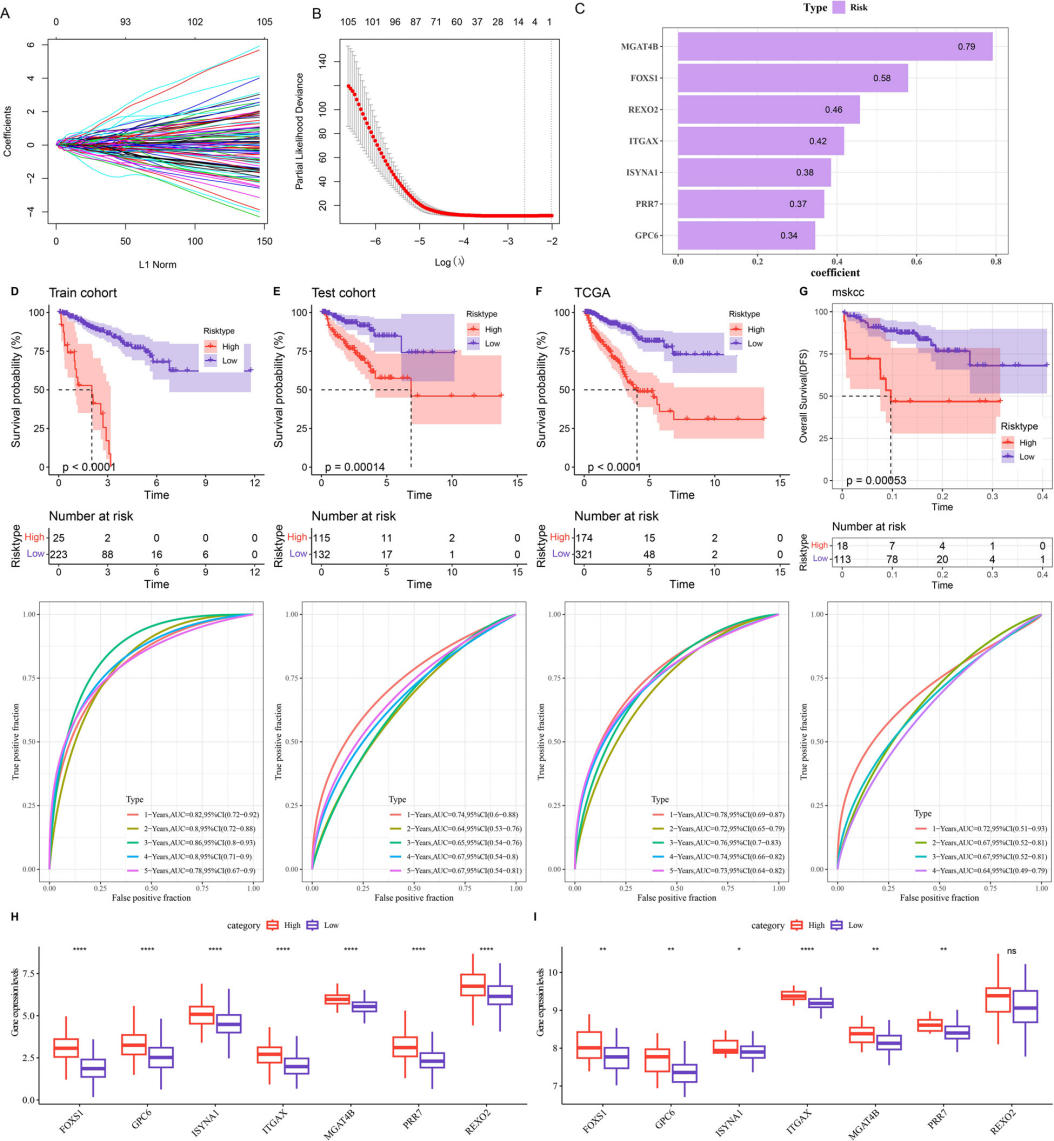
Construction and validation of the risk model. **(A, B)** Changes in the regression coefficients of gene features in the LASSO regression model and the optimal penalty parameter (λ) determined by cross-validation. **(C)** Genes and their coefficients in the risk model. **(D-G)** 1 - 5-year ROC curves of the risk model and differences in PFI between the high-risk and low-risk groups in the training set **(D)**, test set **(E)**, TCGA cohort **(F)**, and MSKCC cohort **(G)**. **(H)** Expression of seven prognostic genes in the TCGA cohort. **(I)** Expression of seven prognostic genes in the MSKCC cohort. ns denotes *p* > 0.05, **p < 0.01, ****p < 0.0001. * means vs. High-risk.


The TCGA-PRAD samples were assigned at the ratio of 5:5 into a training set and a testing set to construct the model. Using the data from the training set, the intersected genes were subjected to univariate Cox proportional hazards regression. Subsequently, LASSO COX and stepwise regression were applied to further shrink the range of genes, and seven genes independently associated with prognosis were screened out for constructing the RiskScore model (**Figures 4A, B**). The formula for this RiskScore model is (**Figure 4C**):

RiskScore = 0.579 * FOXS1 + 0.345 * GPC6 + 0.385 * ISYNA1 + 0.418 * ITGAX + 0.792 * MGAT4B + 0.368 * PRR7 + 0.458 * REXO2

Patients were classified into high- and low-risk group by the optimal cut-off value of the RiskScore. The ROC curve demonstrated that the TCGA cohort, training set, and testing set all exhibited relatively high AUC values at various time points, which verified its good classification accuracy. Moreover, compared with those with a low-risk score, the PFI of patients with a high-risk score was significantly lower (**Figures 4D-F**, *p* < 0.001). The same method was used to validate the MSKCC dataset, and the model also had a relatively high AUC value. There was a significant difference in prognosis between two risk groups (**Figure 4G**). By analyzing the expressions of prognostic genes in the TCGA cohort and the MSKCC cohort, it was found that, except for the *REXO2* gene which showed no significant difference between the two risk groups in the MSKCC dataset, the expressions of the other genes in the low-risk group were all remarkably lower (**Figures 4H-I**, *p* < 0.05).

### 3.5 Correlation between the RiskScore and TIME

**Figure 5:**
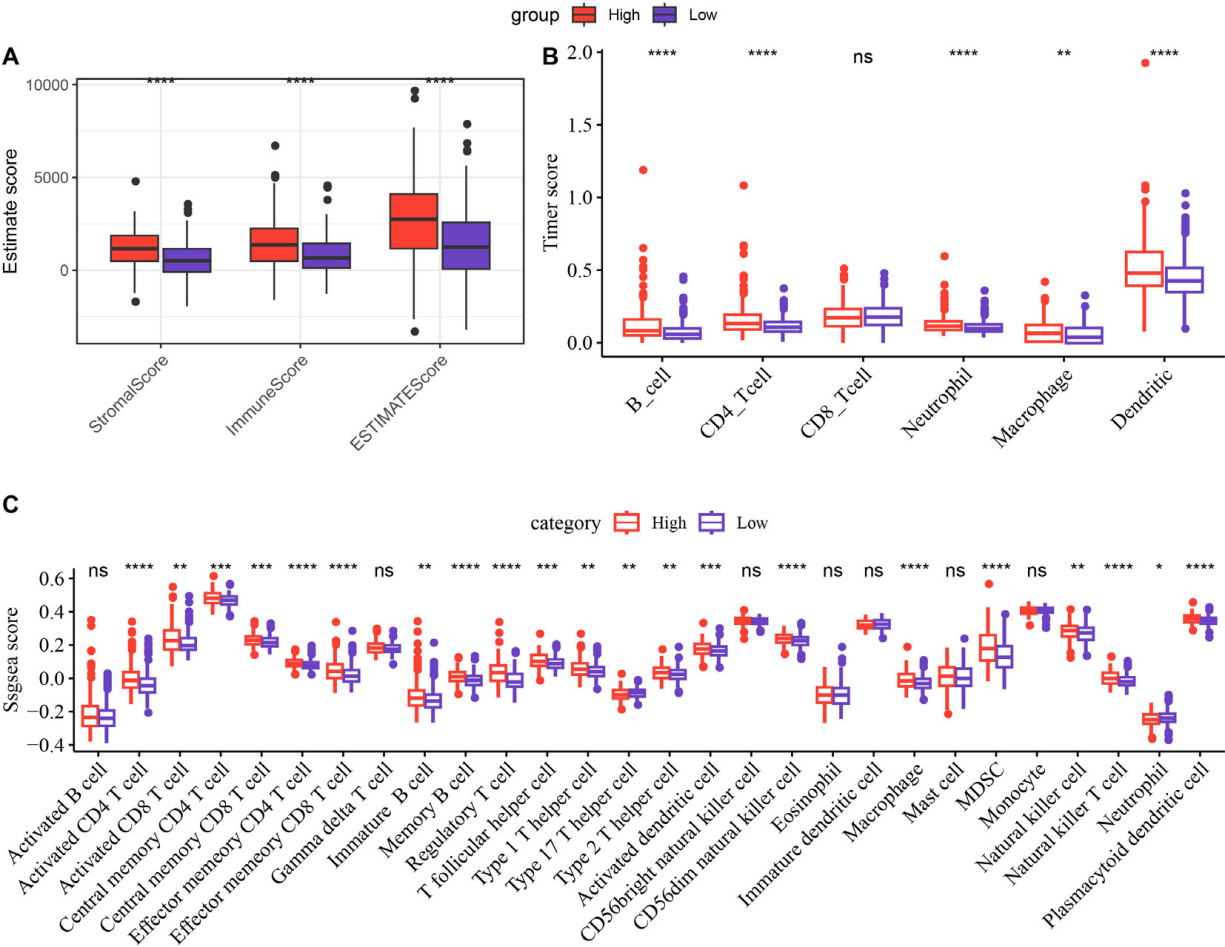
Differences in immune infiltration between the high-risk and low-risk groups. Differences in ESTIMATE **(A)**, Timer **(B)**, and ssGSEA **(C)** scores between the high-risk and low-risk groups in the TCGA cohort. *p < 0.05, **p < 0.01, ***p < 0.001, ****p < 0.0001. ns (abbreviation of "non-significant") means *p* > 0.05.

To analyze the relationship between RiskScore and TIME, different methods were used to calculate the infiltration of immune cells. Analyzing the results of immune cell infiltration assessment by ESTIMATE, it was found that the low-risk group had lower StromalScore, ImmuneScore and ESTIMATEScore than the high-risk group, indicating that the immune infiltration level was lower in the low-risk group (**Figure 5A**, *p* < 0.05). Based on TIMER, the immune cell scores of the TCGA dataset were calculated, and the results showed that the scores of Neutrophil, B_cell, Dendritic, Macrophage, CD4_Tcell were all remarkably lower in the low-risk group (**Figure 5B**, *p* < 0.01). Using ssGSEA functional analysis to score 28 types of immune cells (35), it could be seen that the infiltration scores of multiple immune cells such as myeloid derived suppressor cells (MDSC), activated CD8 T cell, NKT cell, regulatory T cell (Treg), NK cell in the low-risk group were all significantly lower (**Figure 5C**, *p* < 0.05).

### 3.6 Differences in enriched pathways between high-risk and low-risk groups

**Figure 6:**
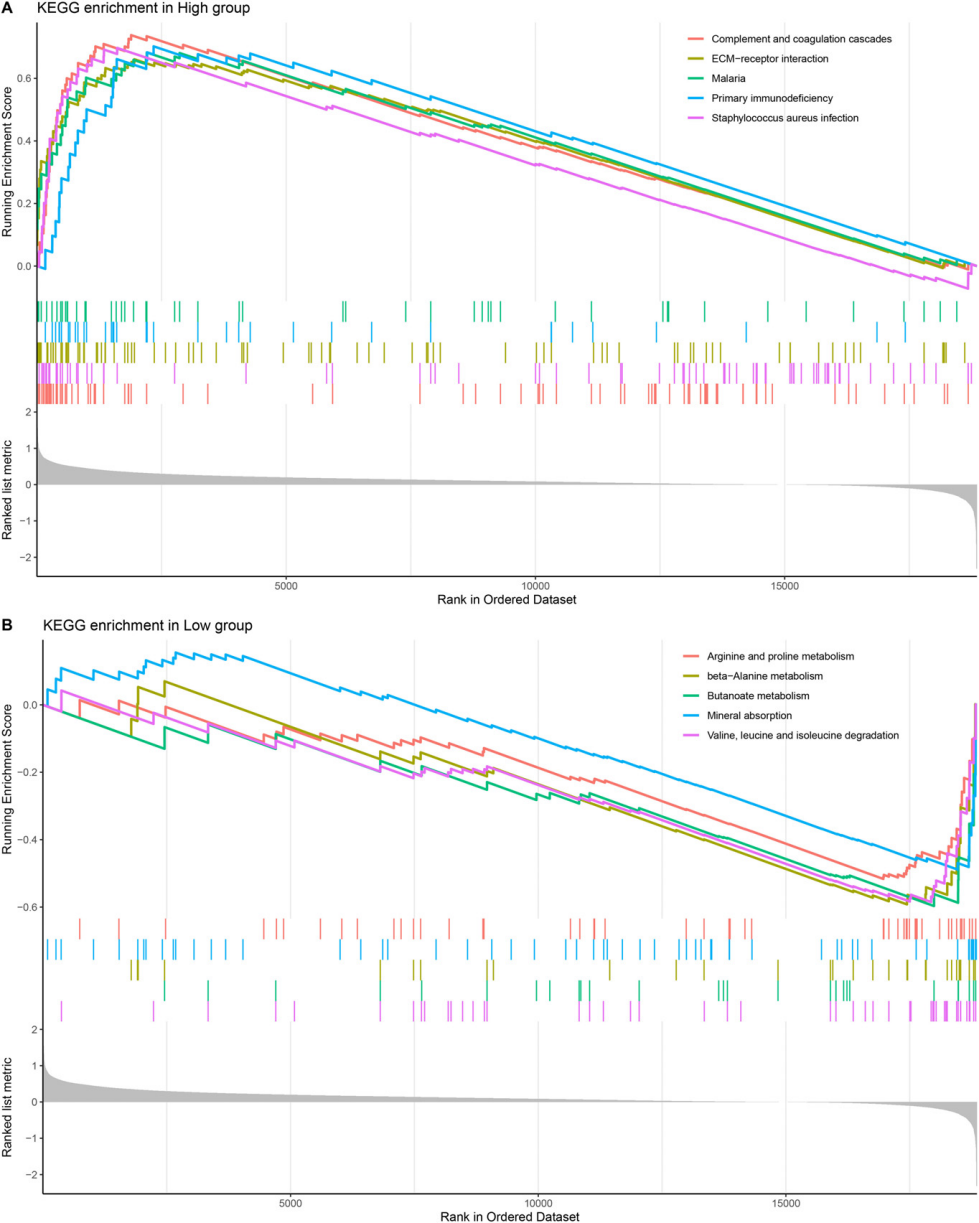
KEGG enrichment pathway analysis. **(A, B)** KEGG enrichment pathway analysis of high-risk and low-risk groups.

The results of KEGG pathway enrichment analysis demonstrated that the high-risk group was significantly enriched in primary immunodeficiency, malaria, complement and coagulation cascades, ECM−receptor interaction, staphylococcus aureus infection pathways (**Figure 6A**). The low-risk group was significantly enriched in arginine and proline metabolism, beta−Alanine metabolism, Butanoate metabolism, mineral absorption and valine, leucine and isoleucine degradation pathways (**Figure 6B**).

### 3.7 Characterization of immune escape potential and immunosuppression in PRAD patients from different risk groups

**Figure 7:**
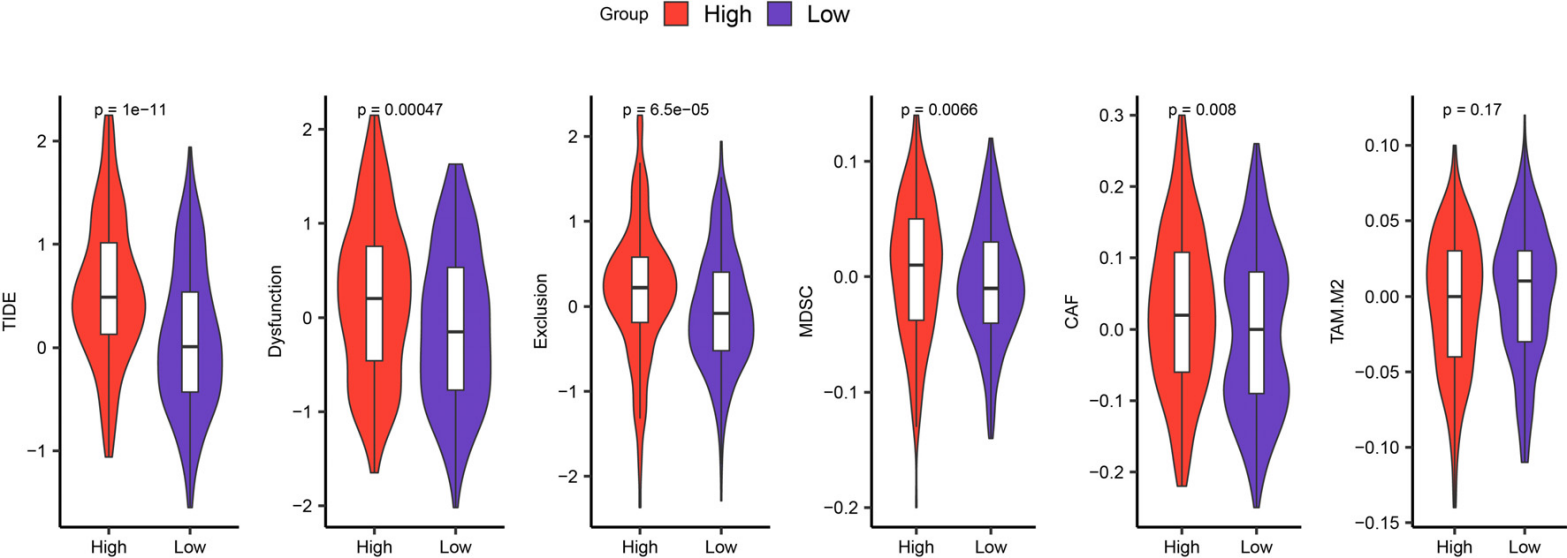
Differences in TIDE scores between high-risk and low-risk groups in TCGA.

TIDE was employed to estimate the potential clinical immunotherapy benefit to the two risk groups. The results demonstrated that in the TCGA cohort, the high-risk group had the highest TIDE score, showing a higher likelihood of immune escape in the high-risk group and less immunotherapy benefit (**Figure 7**, *p* < 0.05). Moreover, in the high-risk group, the scores of dysfunction, exclusion, MDSC, and cancer associated fibroblast (CAF) were significantly higher than those in the low-risk group, which revealed the significant immunosuppressive features and complex mechanisms in the TME (**Figure 7**, *p* < 0.05).

### 3.8 *In vitro* experiments to validate the function of key genes in PRAD cells and the potential role of *ISYNA1*

**Figure 8:**
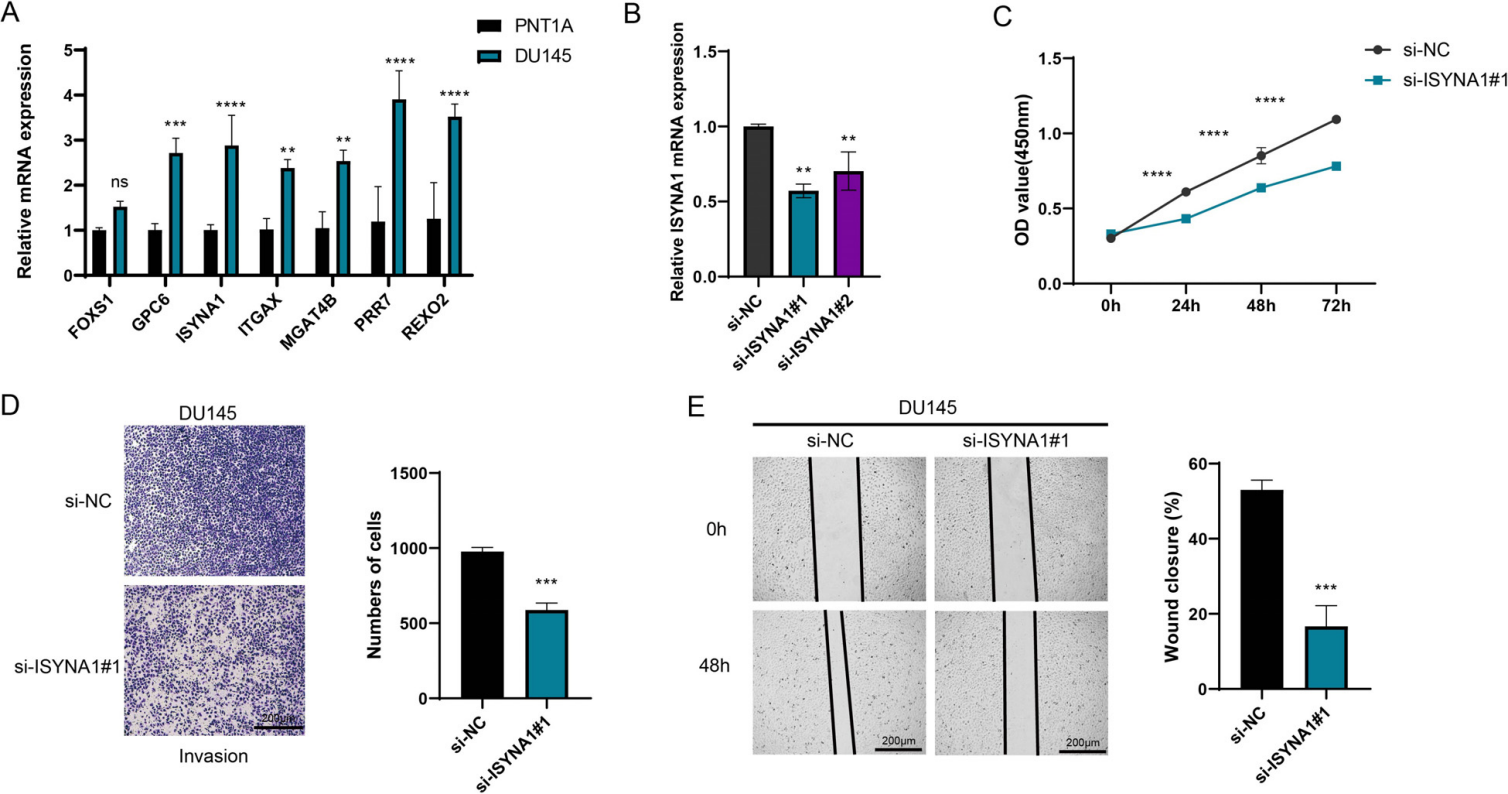
Cell validation results. **(A)** Quantified mRNA level of seven feature genes in PRAD cell line DU145 and the control cell line PNT1A. **(B)** The qRT-PCR to verify the transfection efficiency of ISYNA1 between the si-negative control (si-NC) and si-*ISYNA1* groups. **(C)** Assessment of the proliferation level of DU145 cells after *ISYNA1* silencing based on the CCK-8 assay. **(D)** The results of Transwell assay on the invasion of DU145 cells after the silencing of *ISYNA1*. **(E)** The results of scratch assay on the migration of DU145 cells after the silencing of *ISYNA1*. **p < 0.01, ***p < 0.001, ****p < 0.0001, and ns stands for no significant difference.

Subsequently, the roles of seven characteristic genes in PRAD were validated by *in vitro* experiments. The results showed that all six genes (*GPC6*, *ISYNA1*, *ITGAX*, *MGAT4B*, *PRR7*, and *REXO2*), except *FOXS1*, were significantly highly expressed in PRAD cells (**Figure 8A**, *p* < 0.01). Since *ISYNA1* is highly expressed in DU145 cells and to date, there are few studies on *ISYNA1* in tumor research, especially PRAD. For this reason, we chose to silence *ISYNA1* in order to validate its potential effect on PRAD cell development (**Figure 8B**, *p* < 0.01). Subsequently, we observed a significant decrease in the proliferation, migration and invasion ability of DU145 cells after silencing *ISYNA1* relative to controls (**Figures 8C-E**, *p* < 0.001). These results suggest the potential impact of our NK/T cell-related key gene-based screen on PRAD cell genesis and development.

## 4 Discussion

According to global data in 2022, although the mortality rate of PRAD ranks only eighth, its incidence rate ranks fourth (36), and it has a high recurrence rate, with about one-third of men experiencing recurrence after local treatment, ultimately leading to the generation of malignant cells (37). NK cells are cytotoxic immune cells capable of killing cancer cells in the innate immune system (38). Research has found that cellular communication functions importantly in the TME (39), particularly involving NK/T cells, which are associated with the immune evasion mechanisms of cancers (40). In this study, by analyzing PRAD-related single-cell data, the interaction between NK/T cells and other cells was identified, and a risk model related to communication receptors and ligands was established. These results provided new insights into the immunotherapy of PRAD and opening up new potential pathways for personalized cancer treatment.

The risk model in this study includes a total of seven related molecular markers, namely *FOXS1*, *GPC6*, *ISYNA1*, *ITGAX*, *MGAT4B*, *PRR7*, and *REXO2*. As a type of DNA-binding proteins, Forkhead-box (FOX) family proteins participate in cell growth, embryogenesis, differentiation, and lifespan (41). *FOXS1* has a high expression in pan-cancers, and the gene is linked to a worse surviva. Its overexpression is related to high infiltration levels of tumor-associated macrophages (TAM) and Tregs (42), which is consistent with the result of a higher infiltration level of Tregs in the high-risk group in this study. *GPC6* belongs to the glypican gene family, and its function depends on interactions with cytoplasmic and/or extracellular ligands (43). Chen et al.’s research has confirmed that *GPC2* in the GPC family can promote the progression of PRAD (44). *ISYNA1* encodes an enzyme essential for inositol biosynthesis. Silencing *ISYNA1* can inhibit the growth of tumor cells (45). Jia et al.’s research has demonstrated that it can serve as a biomarker related to pan-cancer immunomodulation prognosis (46). The *in vitro* experiments in this study also confirmed that the knockout of *ISYNA1* would influence both the migration and invasion abilities of PRAD cells. *ITGAX* usually functions as a receptor for the extracellular matrix and is associated with tumor angiogenesis (47, 48). Williams et al.’s research has confirmed that it can act as a susceptibility gene for aggressive PRAD (49). The MGAT4B protein belongs to the glycosyltransferase family and can recognize and bind to both donor and receptor substrates (50). In liver cancer cells, studies have found that the expression of *MGAT4B* is upregulated and it is related to immune evasion (51). PRR7 is a transmembrane adaptor protein (TRAP) and an important organizer and regulator of immune receptor-mediated signal transduction (52). It mediates T cell receptor signaling by assembling the membrane-proximal signalosome (53). *REXO2* is a homologue of the prokaryotic oligoribonuclease existing in human mitochondria and cytoplasm (54). Previous experiments have confirmed that *REXO2* can serve as a biomarker for PRAD (55). The above evidence demonstrates the possible roles that these cell communication ligand-related genes may play in cancer and also implies their potential as biomarkers for PRAD.

Analysis on the correlation between the RiskScore and the immune microenvironment revealed that most of the immune scores in the high-risk group were higher. However, despite the higher scores of NK cells, the scores of Tregs in the high-risk group were also higher. Tregs are a type of T lymphocyte with immunosuppressive functions and functions crucially in the process of the immune system eliminating tumor cells as well as in immune self-tolerance (56). Tregs play an immunosuppressive role in the TIME. Studies have confirmed that Tregs can promote tumor immune evasion through the activation of transforming growth factor-β (TGF-β) mediated by integrin αvβ8 (57, 58). Therefore, although there is a strong immune response in the high-risk group, the high scores of Tregs indicate that tumors may “escape” immune attacks by inducing an immunosuppressive environment. In addition, the biomarkers *FOXS1* and *PRR7* obtained in this study are both closely related to Treg signal transduction or infiltration (42, 53), which further implies that high-risk patients affect the TME through the immune evasion mechanism.

The results of pathway enrichment showed that the pathways in the low-risk group were mainly enriched in metabolic pathways, whereas the high-risk group were mainly enriched in immune-related pathways. This reflects that the high-risk group is linked to immune dysfunction, while the low-risk group is in a relatively healthy state. The TIDE analysis revealed that the exclusion score of the high-risk group was high, suggesting that effector immune cells have difficulty effectively infiltrating into the interior of the tumor, which may be related to physical barriers or chemical repellent factors in the TME. Meanwhile, the score of MDSC in the high-risk group was also relatively high. MDSC is a heterogeneous group of immature bone marrow cells with the potential to effectively target T cells and suppress autoimmune responses (59, 60). Studies have already demonstrated that MDSC can influence the suppression of specific T cell responses in myeloma by inducing the development of Tregs in the TME (61). Combined with the results of immune infiltration, the high score of MDSC indicates that these cells may suppress effector immune responses by influencing Tregs, thereby helping the tumor achieve immune evasion. The increase in the score of CAF further reflects the activity of CAFs. CAF activated by TGF-β is one of the most abundant stromal cell types in almost all solid tumors, and its special role is widely recognized (62). Moreover, TGF-β is closely related to Tregs, NK cells, and immune evasion (63, 64). In conclusion, these characteristics of the high-risk group jointly reveal the tumor’s strategy of enhancing its survival ability through multi-level immune suppression mechanisms, providing important implications for in-depth research on tumor immunotherapy.

The limitations of this study require further consideration and refinement. Firstly, the sample size and representativeness of this study may not fully encapsulate the heterogeneity and diversity of PRAD within the general population, potentially limiting the generalizability of the study results. To address this issue, future research should include larger and more diversified cohorts of PRAD samples, encompassing different stages and subtypes of the disease as well as various ethnic groups. Additionally, the data verification in this study mainly relies on bioinformatics techniques and *in vitro* experiments, lacking the support of *in vivo* experiments. To verify our research results more comprehensively, the construction of relevant animal models is an essential step. Furthermore, the clinical application of the identified biomarkers and risk models needs to be further explored and validated in clinical trials.

## 5 Conclusion

The present study successfully developed a risk model closely related to communication receptors and ligands, marking significant progress in exploring the immune mechanisms of PRAD. The establishment of this model not only deepened our understanding of the complex immune microenvironment of PRAD, but also provided strong theoretical support and practical tools for the formulation of stratified and personalized strategies for PRAD. Furthermore, we confirmed a crucial role of NK/T cells in the development of PRAD, offering new targets and ideas for the progression of novel immunotherapies. In the future, we anticipate enhancing the antitumor activity of NK/T cells by modulating the expression or function of communication receptors and ligands, or designing more precise targeted drugs to inhibit the immune escape mechanisms of tumor cells, thereby further improving the treatment outcomes of PRAD and bringing more effective therapy and better quality of life to patients.

### 5.1 Scope statement

Compared with the benign group, NK/T cells were the cell type with the greatest changes in the tumor group, and their communication intensity was relatively high among all cell types. A RiskScore model was constructed as follows: 0.579*FOXS1 + 0.345*GPC6 + 0.385*ISYNA1 + 0.418*ITGAX + 0.792*MGAT4B + 0.368*PRR7 + 0.458*REXO2 . Analysis of the differences between the two risk groups showed that the level of immune infiltration was higher in the high-risk group, and it was significantly enriched in immune-correlated pathways, while the low-risk group was mainly enriched in metabolism-related pathways. TIDE analysis indicated that the high-risk group had higher immune escape potential. The cellular validation assays have revealed the higher expression of seven biomarkers in PRAD groups. Further, *ISYNA1* knockdown inhibited the migratory and invasive capabilities of PRAD cells. The current research reveals key communication genes in PRAD, offering new possibilities for the exploration of new therapeutic targets.

## Data Availability

The datasets presented in this study can be found in online repositories. The names of the repository/repositories and accession number(s) can be found in the article/**Supplementary Material**.

## Glossary

PCa: prostate cancer
PRAD: prostate adenocarcinoma
mCRPC: metastatic castration-resistant prostate cancer
scRNA-seq: single-cell RNA sequencing
TME: tumor microenvironment
NK: natural killer
TCGA: The Cancer Genome Atlas
FPKM: ragments per kilobase of exon model per million mapped fragments
RNA-Seq: RNA sequencing
TPM: transcripts per million
PFI: progression-free interval
GEO: Gene Expression Omnibus
PCA: principal component analysis
UMAP: uniform manifold approximation and projection
DEGs: differentially expressed gene
LASSO: least absolute shrinkage and selection operator
KM: Kaplan-Meier
ROC: receiver operating characteristic
TIME: tumor immune microenvironment
RPMI: Roswell Park Memorial Institute
FBS: fetal bovine serum
qRT-PCR: quantitative real-time PCR
NCBI: National Center for Biotechnology Information
PBS: phosphate buffered saline
ssGSEA: single sample gene set enrichment analysis
TIIC: tumor infiltrating immune cell
KEGG: kyoto encyclopedia of genes and genomes
TIDE: tumor immune dysfunction and exclusion
AUC: Area Under the Curve
CAF: cancer associated fibroblast
FOX: forkhead-box
TAM: tumor-associated macrophages
Treg: regulatory T cell
TRAP: transmembrane adaptor protein
TGF-β: transforming growth factor-β.

## References

[1] Luo C-G Zhang J An Y-Z Liu X Li S-J Zhang WEI. MAPK9 as a therapeutic target: unveiling ferroptosis in localized prostate cancer progression. Biocell. (2024) 48:771–92. doi: 10.32604/biocell.2024.048878

[2] Xu B Duan H Xie T. The preventive mechanisms and research progress of sulforaphane in relation to prostate cancer. Biocell. (2024) 48:1703–19. doi: 10.32604/biocell.2024.054873

[3] Culp MB Soerjomataram I Efstathiou JA Bray F Jemal A. Recent global patterns in prostate cancer incidence and mortality rates. Eur Urol. (2020) 77:38–52. doi: 10.1016/j.eururo.2019.08.005 31493960

[4] Gandaglia G Leni R Bray F Fleshner N Freedland SJ Kibel A. Epidemiology and prevention of prostate cancer. Eur Urol Oncol. (2021) 4:877–92. doi: 10.1016/j.euo.2021.09.006 34716119

[5] Rawla P. Epidemiology of prostate cancer. World J Oncol. (2019) 10:63–89. doi: 10.14740/wjon1191 31068988 PMC6497009

[6] Berish RB Ali AN Telmer PG Ronald JA Leong HS. Translational models of prostate cancer bone metastasis. Nat Rev Urol. (2018) 15:403–21. doi: 10.1038/s41585-018-0020-2 29769644

[7] Tagawa ST Beltran H Vallabhajosula S Goldsmith SJ Osborne J Matulich D. Anti–prostate-Specific membrane antigen-based radioimmunotherapy for prostate cancer. Cancer. (2010) 116:1075–83. doi: 10.1002/cncr.v116.4s PMC2820151 20127956

[8] Bansal D Reimers MA Knoche EM Pachynski RK. Immunotherapy and immunotherapy combinations in metastatic castration-resistant prostate cancer. Cancers. (2021) 13:334. doi: 10.3390/cancers13020334 33477569 PMC7831137

[9] Yeku O Slovin SF. Immune therapy for prostate cancer. Cancer J. (2016) 22:334–41. doi: 10.1097/PPO.0000000000000223 PMC5117426 27749327

[10] Teo MY Rathkopf DE Kantoff P. Treatment of advanced prostate cancer. Annu Rev Med. (2019) 70:479–99. doi: 10.1146/annurev-med-051517-011947 PMC6441973 30691365

[11] Wang B Niu X. A network medical framework based on inflammatory genes to identify drug candidates for abdominal aortic aneurysms. Curr Mol Pharmacol. (2023) 17:e170523216998. doi: 10.2174/1874467217666230517104426 37198994

[12] Meng F Li L Lu F Yue J Liu Z Zhang W. Overexpression of TIGIT in NK and T cells contributes to tumor immune escape in myelodysplastic syndromes. Front Oncol. (2020) 10. doi: 10.3389/fonc.2020.01595 PMC7438899 32903786

[13] Lee MY Robbins Y Sievers C Friedman J Abdul Sater H Clavijo PE. Chimeric antigen receptor engineered NK cellular immunotherapy overcomes the selection of T-cell escape variant cancer cells. J immunotherapy Cancer. (2021) 9:e002128. doi: 10.1136/jitc-2020-002128 PMC7986659 33741731

[14] Nguyen LT Lo CS Fyrsta M Nie J Yam JY Yen PH. Expansion of lymphocytes from prostatic adenocarcinoma and adjacent nonmalignant tissue. Prostate Cancer. (2022) 2022:6499344. doi: 10.1155/2022/6499344 35754788 PMC9225894

[15] Kim T-J Kim M Kim HM Lim SA Kim E-O Kim K. Homotypic NK cell-to-cell communication controls cytokine responsiveness of innate immune NK cells. Sci Rep. (2014) 4:7157. doi: 10.1038/srep07157 25475707 PMC4256668

[16] Shao X Lu X Liao J Chen H Fan X. New avenues for systematically inferring cell-cell communication: through single-cell transcriptomics data. Protein Cell. (2020) 11:866–80. doi: 10.1007/s13238-020-00727-5 PMC7719148 32435978

[17] Ma F Zhang S Song L Wang B Wei L Zhang F. Applications and analytical tools of cell communication based on ligand-receptor interactions at single cell level. Cell Bioscience. (2021) 11:121. doi: 10.1186/s13578-021-00635-z 34217372 PMC8254218

[18] Shahrajabian MH Sun W. Survey on multi-omics, and multi-omics data analysis, integration and application. Curr Pharm Anal. (2023) 19:267–81. doi: 10.2174/1573412919666230406100948

[19] Hao YH Stuart T Kowalski MH Choudhary S Hoffman P Hartman A. Dictionary learning for integrative, multimodal and scalable single-cell analysis. Nat Biotechnol. (2024) 42:293–304. doi: 10.1038/s41587-023-01767-y 37231261 PMC10928517

[20] Korsunsky I Millard N Fan J Slowikowski K Zhang F Wei K. Fast, sensitive and accurate integration of single-cell data with Harmony. Nat Methods. (2019) 16:1289–96. doi: 10.1038/s41592-019-0619-0 PMC6884693 31740819

[21] Xu X Huang Y Han X. Single-nucleus RNA sequencing reveals cardiac macrophage landscape in hypoplastic left heart syndrome. Congenital Heart Dis. (2024) 19:233–46. doi: 10.32604/chd.2024.050231

[22] Jin S Guerrero-Juarez CF Zhang L Chang I Ramos R Kuan C-H. Inference and analysis of cell-cell communication using CellChat. Nat Commun. (2021) 12:1088. doi: 10.1038/s41467-021-21246-9 33597522 PMC7889871

[23] Ritchie ME Phipson B Wu D Hu Y Law CW Shi W. limma powers differential expression analyses for RNA-sequencing and microarray studies. Nucleic Acids Res. (2015) 43:e47. doi: 10.1093/nar/gkv007 25605792 PMC4402510

[24] Kumar M Sonker PK Saroj A Jain A Bhattacharjee A Saroj RK. Parametric survival analysis using R: Illustration with lung cancer data. Cancer Rep (Hoboken). (2020) 4:e1210. doi: 10.1002/cnr2.121 PMC7941555 32794636

[25] Simon N Friedman J Hastie T Tibshirani R. Regularization paths for cox's proportional hazards model via coordinate descent. J Stat software. (2011) 39:1–13. doi: 10.18637/jss.v039.i05 PMC4824408 27065756

[26] Zhang X Chao P Zhang L Xu L Cui X Wang S. Single-cell RNA and transcriptome sequencing profiles identify immune-associated key genes in the development of diabetic kidney disease. Front Immunol. (2023). 14:1030198. doi: 10.3389/fimmu.2023.1030198 37063851 PMC10091903

[27] Song ZG Yu JB Wang MM Shen WT Wang CC Lu TY. CHDTEPDB: transcriptome expression profile database and interactive analysis platform for congenital heart disease. Congenital Heart Dis. (2023) 18:693–701. doi: 10.32604/chd.2024.048081

[28] Hänzelmann S Castelo R Guinney J. GSVA: gene set variation analysis for microarray and RNA-Seq data. BMC Bioinf. (2013) 14:7. doi: 10.1186/1471-2105-14-7 PMC3618321 23323831

[29] Yoshihara K Shahmoradgoli M Martínez E Vegesna R Kim H Torres-Garcia W. Inferring tumour purity and stromal and immune cell admixture from expression data. Nat Commun. (2013) 4:2612. doi: 10.1038/ncomms3612 24113773 PMC3826632

[30] Yu G Wang L-G Han Y He Q-Y. clusterProfiler: an R package for comparing biological themes among gene clusters. Omics: A J Integr Biol. (2012) 16:284–7. doi: 10.1089/omi.2011.0118 PMC3339379 22455463

[31] Wang S Xie C Hu H Yu P Zhong H Wang Y. iTRAQ-based proteomic analysis unveils NCAM1 as a novel regulator in doxorubicin-induced cardiotoxicity and DT-010-exerted cardioprotection. Curr Pharm Anal. (2025) 20:966–77. doi: 10.2174/0115734129331758241022113026

[32] Trick AY Chen FE Schares JA Freml BE Lor P Yun Y. High resolution estimates of relative gene abundance with quantitative ratiometric regression PCR (qRR-PCR). Analyst. (2021) 146:6463–9. doi: 10.1039/D1AN01397A PMC8627783 34605831

[33] Zhang X Jin M Yao X Liu J Yang Y Huang J. Upregulation of lncRNA WT1-AS inhibits tumor growth and promotes autophagy in gastric cancer via suppression of PI3K/akt/mTOR pathway. Curr Mol Pharmacol. (2024) 17:e18761429318398. doi: 10.2174/0118761429318398240918063450 39592900

[34] Wang J Cai Y Sheng Z Dong Z. EGFR inhibitor CL-387785 suppresses the progression of lung adenocarcinoma. Curr Mol Pharmacol. (2023) 16:211–6. doi: 10.2174/1874467215666220329212300 35352671

[35] Charoentong P Finotello F Angelova M Mayer C Efremova M Rieder D. Pan-cancer immunogenomic analyses reveal genotype-immunophenotype relationships and predictors of response to checkpoint blockade. Cell Rep. (2017) 18:248–62. doi: 10.1016/j.celrep.2016.12.019 28052254

[36] Bray F Laversanne M Sung H Ferlay J Siegel RL Soerjomataram I. Global cancer statistics 2022: GLOBOCAN estimates of incidence and mortality worldwide for 36 cancers in 185 countries. CA Cancer J Clin. (2024) 74:229–63. doi: 10.3322/caac.21834 38572751

[37] Chandrasekar T Yang JC Gao AC Evans CP. Urology, Mechanisms of resistance in castration-resistant prostate cancer (CRPC). Trans andrology Urol. (2015) 4:365–80. doi: 10.3978/j.issn.2223-4683.2015.05.02 PMC4708226 26814148

[38] Myers JA Miller JS. Exploring the NK cell platform for cancer immunotherapy. Nat Rev Clin Oncol. (2021) 18:85–100. doi: 10.1038/s41571-020-0426-7 32934330 PMC8316981

[39] Maia J Caja S Strano Moraes MC Couto N Costa-Silva B. Exosome-based cell-cell communication in the tumor microenvironment. Front Cell Dev Biol. (2018) 6. doi: 10.3389/fcell.2018.00018 PMC5826063 29515996

[40] Anichini A Perotti VE Sgambelluri F Mortarini R. Immune escape mechanisms in non small cell lung cancer. Cancers. (2020) 12:3605. doi: 10.3390/cancers12123605 33276569 PMC7761620

[41] Katoh M Igarashi M Fukuda H Nakagama H Katoh M. Cancer genetics and genomics of human FOX family genes. Cancer Lett. (2013) 328:198–206. doi: 10.1016/j.canlet.2012.09.017 23022474

[42] Liu Y Tu M Wang L. Pan-cancer analysis predicts FOXS1 as a key target in prognosis and tumor immunotherapy. Int J Gen Med. (2022) 15:2171–85. doi: 10.2147/IJGM.S354195 PMC8887970 35241932

[43] Dinccelik−Aslan M Gumus−Akay G Elhan AH Unal E Tukun A. Diagnostic and prognostic significance of glypican 5 and glypican 6 gene expression levels in gastric adenocarcinoma. Mol Clin Oncol. (2015) 3:584–90. doi: 10.3892/mco.2015.486 PMC4471538 26137271

[44] Chen S Liao J Li J Wang S. GPC2 promotes prostate cancer progression via MDK-mediated activation of PI3K/AKT signaling pathway. Funct Integr Genomics. (2024) 24:127. doi: 10.1007/s10142-024-01406-y 39014225 PMC11252201

[45] Koguchi T Tanikawa C Mori J Kojima Y Matsuda K. Regulation of myo-inositol biosynthesis by p53-ISYNA1 pathway. Int J Of Oncol. (2016) 48:2415–24. doi: 10.3892/ijo.2016.3456 27035231

[46] Jia Z Wan X. ISYNA1: an immunomodulatory-related prognostic biomarker in colon adenocarcinoma and pan-cancer. Front Cell Dev Biol. (2022) 10. doi: 10.3389/fcell.2022.792564 PMC8883116 35237596

[47] Sui Y Lu K Fu L. Prediction and analysis of novel key genes ITGAX, LAPTM5, SERPINE1 in clear cell renal cell carcinoma through bioinformatics analysis. PeerJ. (2021) 9:e11272. doi: 10.7717/peerj.11272 33976979 PMC8063882

[48] Wang J Yang L Liang F Chen Y Yang G. Integrin alpha x stimulates cancer angiogenesis through PI3K/Akt signaling–mediated VEGFR2/VEGF-A overexpression in blood vessel endothelial cells. J Cell Biochem. (2019) 120:1807–18. doi: 10.1002/jcb.v120.2 30873824

[49] Williams KA Lee M Hu Y Andreas J Patel SJ Zhang S. A systems genetics approach identifies CXCL14, ITGAX, and LPCAT2 as novel aggressive prostate cancer susceptibility genes. PloS Genet. (2014) 10:e1004809. doi: 10.1371/journal.pgen.1004809 25411967 PMC4238980

[50] Nagae M Hirata T Tateno H Mishra SK Manabe N Osada N. Discovery of a lectin domain that regulates enzyme activity in mouse N-acetylglucosaminyltransferase-IVa (MGAT4A). Commun Biol. (2022) 5:695. doi: 10.1038/s42003-022-03661-w 35854001 PMC9296478

[51] Zeng S Ding T. IDDF2023-ABS-0230 The role of aberrant N-Glycan branching regulated by MGAT4B in anti-tumor immunity in hepatocellular carcinoma. J Gut. (2023) 72:A47–7. doi: 10.1136/gutjnl-2023-IDDF.33

[52] Hrdinka M Dráber P Štěpánek O Ormsby T Otáhal P Angelisová P. PRR7 is a transmembrane adaptor protein expressed in activated T cells involved in regulation of T cell receptor signaling and apoptosis *. J Biol Chem. (2011) 286:19617–29. doi: 10.1074/jbc.M110.175117 PMC3103341 21460222

[53] Kravchick DO Karpova A Hrdinka M Lopez-Rojas J Iacobas S Carbonell AU. Synaptonuclear messenger PRR7 inhibits c-Jun ubiquitination and regulates NMDA-mediated excitotoxicity. EMBO J. (2016) 35:1923–34. doi: 10.15252/embj.201593070 PMC5007554 27458189

[54] Szewczyk M Malik D Borowski LS Czarnomska, Sylwia D Kotrys AV Klosowska-Kosicka K. Human REXO2 controls short mitochondrial RNAs generated by mtRNA processing and decay machinery to prevent accumulation of double-stranded RNA. Nucleic Acids Res. (2020) 48:5572–90. doi: 10.1093/nar/gkaa302 PMC7261184 32365187

[55] Hua X Ge S Chen J Zhang L Tai S Liang C. Effects of RNA binding proteins on the prognosis and Malignant progression in prostate cancer. Front Genet. (2020) 11. doi: 10.3389/fgene.2020.591667 PMC7606971 33193734

[56] Zou W. Regulatory T cells, tumour immunity and immunotherapy. Nat Rev Immunol. (2006) 6:295–307. doi: 10.1038/nri1806 16557261

[57] Lainé A Labiad O Hernandez-Vargas H This S Sanlaville A Léon S. Regulatory T cells promote cancer immune-escape through integrin αvβ8-mediated TGF-β activation. Nat Commun. (2021) 12:6228. doi: 10.1038/s41467-021-26352-2 34711823 PMC8553942

[58] Oleinika K Nibbs RJ Graham GJ Fraser AR. Suppression, subversion and escape: the role of regulatory T cells in cancer progression. Clin Exp Immunol. (2012) 171:36–45. doi: 10.1111/j.1365-2249.2012.04657.x PMC3530093 23199321

[59] De Cicco P Ercolano G Ianaro A. The new era of cancer immunotherapy: targeting myeloid-derived suppressor cells to overcome immune evasion. Front Immunol. (2020) 11. doi: 10.3389/fimmu.2020.01680 PMC7406792 32849585

[60] Cripps JG Gorham JD. MDSC in autoimmunity. Int Immunopharmacol. (2011) 11:789–93. doi: 10.1016/j.intimp.2011.01.026 PMC3109222 21310255

[61] Malek E de Lima M Letterio JJ Kim B-G Finke JH Driscoll JJ. Myeloid-derived suppressor cells: The green light for myeloma immune escape. Blood Rev. (2016) 30:341–8. doi: 10.1016/j.blre.2016.04.002 PMC6411302 27132116

[62] Calon A Tauriello DVF Batlle E. TGF-beta in CAF-mediated tumor growth and metastasis. Semin Cancer Biol. (2014) 25:15–22. doi: 10.1016/j.semcancer.2013.12.008 24412104

[63] Tauriello DVF Sancho E Batlle E. Overcoming TGFβ-mediated immune evasion in cancer. Nat Rev Cancer. (2022) 22:25–44. doi: 10.1038/s41568-021-00413-6 34671117

[64] Beck C Schreiber H Rowley DA. Role of TGF-β in immune-evasion of cancer. Microscopy Res technique. (2001) 52:387–95. doi: 10.1002/1097-0029(20010215)52:4<387::AID-JEMT1023>3.0.CO;2-W 11170297

