# Supplementary material for: Identification of the gene signatures related to NK/T cell communication to evaluate the tumor microenvironment and prognostic outcomes of patients with prostate adenocarcinoma
Source: Front Immunol. 2025 Apr 16;16:1564784. doi: 10.3389/fimmu.2025.1564784 (PMC12041053; doi:10.3389/fimmu.2025.1564784)
Supplement: Supplementary file 1 [file Table1.docx]

Supplementary Table 1. Primer sequences used in this study.

| Gene | Accession No. | Primers (5’-3’) | |
| --- | --- | --- | --- |
|  |  | Forward | Reverse |
| *FOXS1* | NM_004118 | CCACAACTGAGCCAACCAAGC | GTAGCGGTAGATGCCACTGAGG |
| *GPC6* | NM_005708 | CACTCGCTCTCGCTTGTCCAT | GCTGCTGCCTTGCTCCTCTA |
| *ISYNA1* | NM_016368 | ACTACTACGGCTCGCTGACTCA | GGTTCTCGGCTGTGTCGTTGA |
| *ITGAX* | NM_000887 | GACCAGCAAGACCACCTTCCAG | CACACTCACGACCGACACCTTC |
| *MGAT4B* | NM_014275 | GGTGAGCACGAGCCTGAAGACA | TGGAAGAAGCGGAAGCGGATGA |
| *PRR7* | NM_030567 | GTGGAGAGGAGGAAGCGGAACT | AGCAGCAGAGCAGGACGATGA |
| *REXO2* | NM_015523 | CTGGAGGCAACTTCTGGTGGTT | TTCGTGTGCTGTCTGCTTGAGT |
| *GAPDH* | NM_002046 | GTCTCCTCTGACTTCAACAGCG | ACCACCCTGTTGCTGTAGCCAA |
